# Updates on Breast Cancer

**DOI:** 10.3390/cancers15225392

**Published:** 2023-11-13

**Authors:** Filippo Pesapane, Luca Nicosia, Enrico Cassano

**Affiliations:** Breast Imaging Division, IEO European Institute of Oncology IRCCS, 20141 Milan, Italy; luca.nicosia@ieo.it (L.N.); enrico.cassano@ieo.it (E.C.)

This collection of 18 articles, comprising 12 original studies, 1 systematic review, and 5 reviews, is a collaborative effort by distinguished experts in breast cancer research, and it has been edited by Dr. Enrico Cassano and Dr. Filippo Pesapane, who both work at an international breast cancer referral center [1].

Breast cancer, a globally prevalent malignancy primarily afflicting women [2], remains a pivotal focus of medical research and innovation. In our Special Issue, titled “Updates on Breast Cancer”, we present the latest developments in breast cancer diagnostics, treatments, and patient care, offering an in-depth exploration of the multifaceted nature of breast cancer and the way in which science is transforming this field.

Nowadays, though precision diagnosis and personalized medicine are cornerstones of effective breast cancer management, achieving consistent and reproducible diagnostic assessments remains a challenge. Cserni B. et al. [3] introduce a groundbreaking methodology known as the ONEST (Observers Needed to Evaluate Subjective Tests) analysis. By determining the optimal number of observers required for reliable tumor-infiltrating lymphocyte categorization, ONEST has the potential to revolutionize diagnostic accuracy, providing a more robust and consistent framework for assessing breast cancer pathology.

Precision diagnosis also allows clinicians to understand the molecular intricacies of breast cancer. Monteiro F.L. et al. [4] performed a meticulous analysis that unveils the enigmatic role played by SETD7, a lysine N-methyltransferase, in breast cancer. Among her notable findings is the correlation between high SETD7 expression and worse recurrence-free survival in the basal-like subtype, underscoring this molecule’s clinical significance as a potential treatment-predictive marker.

The study of Nicosia L. et al. [5] introduces a novel nomogram aimed at predicting the likelihood of upstaging low-grade ductal carcinoma in situ in patients who have previously undergone vacuum-assisted breast biopsy, followed by surgical excision. This innovative tool leverages radiological and pathological criteria to provide a tailored framework for making treatment decisions. By identifying patients with a low risk of upstaging to infiltrating carcinomas, this nomogram has the potential to reduce overtreatment and improve patient outcomes, once again showing that personalized medicine is increasingly paramount in breast cancer care.

Radiomics and artificial intelligence (AI) are also driving significant advances in precision diagnosis. Petrillo et al. [6] launch a comprehensive investigation, leveraging radiomics features derived from contrast-enhanced mammography to predict various histological outcomes.

Radiomics, the extraction and analysis of quantitative features from medical images, holds promise in terms of providing valuable information beyond what the human eye can perceive [7,8,9]. The results of Petrillo et al.’s study rested on the analysis of a staggering 837 textural metrics that demonstrate an accuracy of 88.98%, enabling the differentiation of malignant and benign lesions. Beyond this distinction, the study attempted to predict histological grading, the presence of hormone receptors, and the status of human epidermal growth factor receptor 2 (HER2) in breast cancer patients.

As we continue to harness the capabilities of radiomics and AI, we move closer to developing more effective breast cancer management practices, offering hope and improved prospects for afflicted women.

Mohammad Alkhaleefah M. et al. [10] present an innovative deep learning model named Connected-SegNets. This model is engineered for the precise segmentation of breast tumors from X-ray images, incorporating skip connections between layers, thus replacing the conventional loss function with intersection over union to fortify robustness against noise during training.

These findings all highlight the immense potential of radiomics and AI in the realm of breast cancer care [7,8,9]. However, while the integration of AI into radiology is revolutionizing breast cancer diagnosis, continuing to focus on the patient and preserving the doctor–patient relationship is crucial. Derevianko et al.’s study [11] delves into the impact of AI on doctor–patient communication, particularly within the context of cancer diagnosis. Their systematic review emphasizes the need for transparent and informative communication to establish patient trust in AI-driven diagnostic processes, ultimately improving healthcare interactions. Ad it remains uncertain to what extent and under which conditions the general population will embrace the use of AI [12], this study highlights the need to conduct larger-scale research to better understand women’s demands and concerns regarding the potential applications of AI in breast cancer care.

In addition to these improvements in breast cancer diagnosis, advances in postoperative surveillance have significantly contributed to the improved survival rates identified in breast cancer patients. Yang L. et al. [13] present a multicenter real-world study conducted across medical centers in China. Their study investigates the prognostic value of intensive postoperative bone scans for patients with breast cancer and bone metastasis. The findings provide compelling evidence of the benefits of this screening method, showcasing its potential to extend both overall survival and overall survival after bone metastasis.

Despite such improvement, in a breast cancer scenario, triple-negative breast cancer (TNBC) remains a formidable clinical challenge due to its limited therapeutic options. Kholod O. et al. [14] decode the immune-related gene signatures that hold the key to predicting chemoimmunotherapy outcomes in TNBC patients, analyzing a vast dataset encompassing 422 patients across 24 studies. Through an algorithmic approach, they categorize patients into 12 homogenous subgroups based on various parameters, including tumor mutational burden, relapse status, tumor cellularity, menopausal status, and tumor stage.

A comprehensive analysis of the clinical utility of genomic tests in breast cancer care is provided by Galland et al. [15], who explore the clinical utility of genomic tests evaluating homologous recombination repair deficiency in breast cancer treatment decisions. Moreover, Safe S. et al. [16] show the roles played by the aryl hydrocarbon receptor and its ligands in breast cancer progression and the potentialities for homologous recombination repair deficiency in early and metastatic breast cancer. Finally, Valenzuela-Palomo et al. [17] delve into the impacts of these variants on splicing, a crucial step in gene expression regulation. Their use of minigene assays to analyze 16 PALB2 variants at intron/exon boundaries reveals that 12 of these variants disrupt splicing, with 6 variants being classified as likely pathogenic in nature. This study offers essential insights into the clinical management of carrier patients and their families, enabling tailored prevention and therapy protocols.

Our Special Issue exceeds the traditional boundaries of breast cancer research, exploring a diverse array of topics that all enrich our understanding of this complex disease. From the characterization of circulating tumor cells using cutting-edge technology like the Parsortix^®^ PC1 System [18] to investigating the clinical landscape of HER2-Low breast cancer [19], these studies broaden our horizons. With regard to radiation therapy, which is a crucial component of breast cancer treatment, Riaz et al. [20] analyze recent advances in optimizing radiation therapy decisions for early invasive breast cancer. Their exploration of strategies to identify patients who may benefit from tailored radiation therapy regimens shows a commitment to enhancing patient care and outcomes. Lastly, Zahari et al. [21] provide a review of the role played by the cancer cell secretome in breast cancer progression, explaining how the secretome shapes the tumor microenvironment, influences treatment resistance, and offer insights into potential therapeutic strategies targeting its components.

In conclusion, our Special Issue, titled “Updates on Breast Cancer,” shows the remarkable progress taking place within the field of breast cancer research. Technological innovations like radiomics and AI, together with the collective efforts of dedicated researchers, have paved the way for precision diagnosis, enhanced treatment strategies, and more personalized patient care. As we journey through this compendium of research, we are reminded that the pursuit of knowledge and innovation is limitless. The future of breast cancer care is being shaped right now, guided by the dedication and unwavering commitment of the scientific community. We invite our readers to help us to embrace these advancements, as we look forward to a brighter and more promising future in the battle against breast cancer.
